# The complete chloroplast genome of *Vaccinium henryi* Hemsl. 1889 (Ericaceae)

**DOI:** 10.1080/23802359.2024.2444603

**Published:** 2024-12-21

**Authors:** Chunfeng Ge, Gangqiang Dong, Yanqing Jiang, Liangliang Tian, Hong Yu, Qilong Zeng

**Affiliations:** a Jiangsu Key Laboratory for the Research and Utilization of Plant Resources, Institute of Botany, Jiangsu Province and Chinese Academy of Sciences (Nanjing Botanical Garden Mem. Sun Yat-Sen), Nanjing, China; b Amway (China) Botanical R&D Center, Wuxi, China

**Keywords:** *Vaccinium henryi*, chloroplast genome, sequencing, phylogenetic analysis

## Abstract

*Vaccinium henryi* Hemsl. 1889 is an endemic deciduous shrub in China, belonging to the family Ericaceae. In this study, the first complete chloroplast genome of *V. henryi* was assembled and annotated. The genome was 176,339 bp in size containing a large single-copy region of length 106,115 bp, a small single-copy region of length 3022 bp, and a pair of inverted repeat regions of 33,601 bp each. It contained 139 genes, including 91 protein-coding genes, 40 tRNA genes, and 8 rRNA genes. Phylogenetic analysis exhibited *that V. henryi* and *V. oldhamii* were phylogenetic closely related. The chloroplast genome of *V. henryi* would provide valuable information for phylogenetic and evolutionary research on genus *Vaccinium.*

## 1. Introduction

*Vaccinium henryi* Hemsl. 1889 is a member of the genus *Vaccinium* (family Ericaceae). It is an endemic species mainly distributed in Anhui, Fujian, Gansu, Guizhou, Hubei, Hunan, Jiangxi, Shanxi, Sichuan, and Zhejiang provinces of China (Fang Huang, 1997). The plant is about 1–3 m tall, and grows in forests, and thickets on mountain slopes at altitudes from 700 to 2100 m. *V. henryi* is characterized by extremely short pedicel, usually about 1 mm in length or nearly sessile, the young foliage and stems are densely covered with short hair. It flowers from June to July, and the yellow-green flowers have bell-shaped corolla with about 10 stamens (Fang and Stevens 2005).

Interspecific introgression is an important way to obtain new traits in blueberry breeding. *V. henryi* is a native species that has a relatively large fruit size (7–9 mm), and a late fruit ripening time (September to October) which are important characteristics for breeding late-maturing blueberry varieties compared with other wild *Vaccinium* species. In order to speed up the blueberry breeding process, chloroplast genome sequencing would provide many important DNA molecular markers that are useful in species relationships, population genetic diversity and genetic map construction (Holá et al. 2017; Miao et al. 2022). However, the complete chloroplast genome of *V. henryi* has not been evaluated. The primary objectives of this study were to characterize the chloroplast genome sequence of *V. henryi* and to investigate its physiology, and phylogenetic relationship in *Vaccinium*.

## 2. Materials and methods

**Figure 1.**
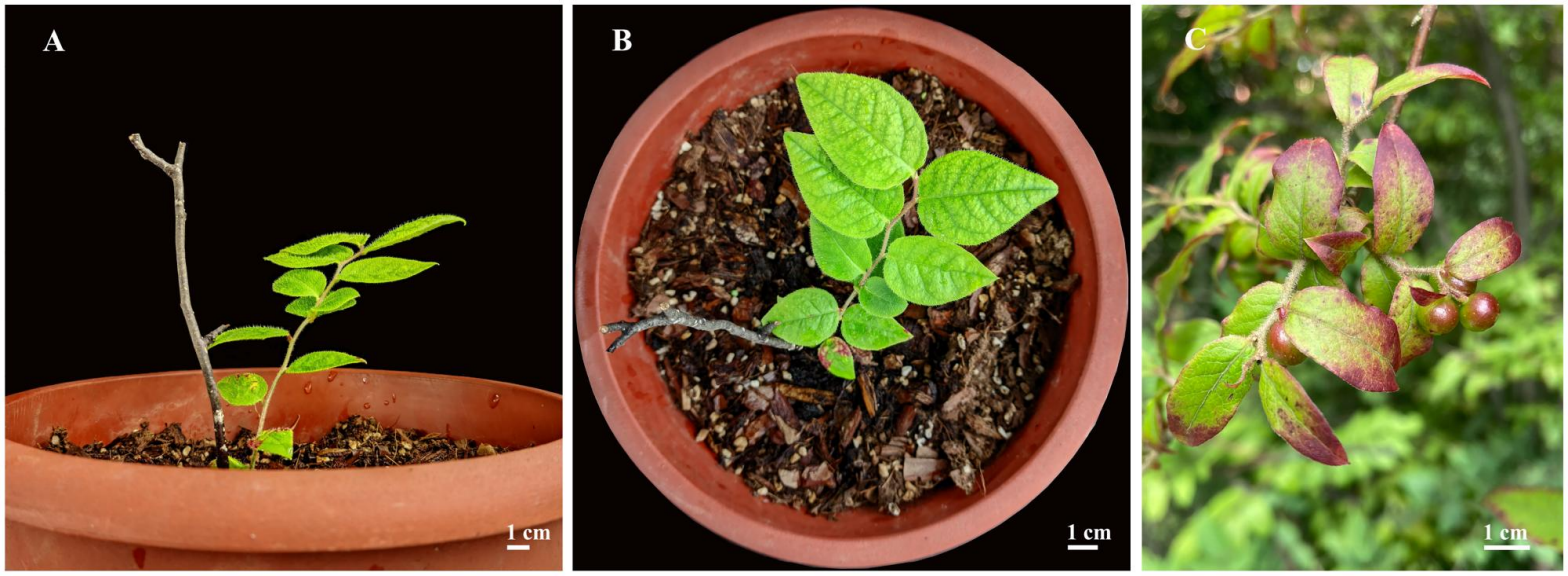
Reference images of *Vaccinium henryi* Hemsl. 1889. The young leaves and stems of *V. henryi* are densely covered with short hair, leaves are usually ovate or ovate-oblong characterized with a sharp apex and a broad wedge-shaped base. The fruits are spherical, slightly oblate, 7-9 mm in diameter, turns purple-black when ripe. (A) Side view of branch and (B) Top view of branch (both images were taken by Chunfeng Ge at Nanjing Botanical Garden Mem. Sun Yat-Sen, Nanjing, China (N32°3’44.2", E118°50’37.0")), (C) Fruits during veraison period in the wild (image was taken by Qilong Zeng at Enshi, China (E108°33′8.3″, N30°21′39.5″)).

The fresh leaf sample of *V. henryi* was collected from Nanjing Botanical Garden Mem. Sun Yat-Sen, Nanjing, China (N32°3’44.2", E118°50’37.0"). The voucher specimen (Accession No. CNBG_2023_WGYJ) was deposited at the Herbarium of Nanjing Botanical Garden, Memorial Sun Yat-sen (http://www.jib.ac.cn/, Chunfeng Ge, gechunfeng@jib.ac.cn) (Figure 1). Total genomic DNA was extracted from a 100 mg sample with a genomic DNA extraction kit (Tiangen Biotech, Beijing, China) and used to construct a cDNA library, and sequenced on Illumina NovaSeq 6000 platform (Illumina Lnc., San Diego, CA) with 150 bp paired-end reads length. A total of 6538.3 Mb of clean data was obtained after quality control filtering. The complete chloroplast genome was assembled using GetOrganelle 1.6.4 (Jin et al. 2020) and the genes were annotated with the GeSeq tool (Tillich et al. 2017). Manual corrections of genes for start/stop codons and for intron/exon boundaries were performed in SnapGene Viewer (GSL BioTech LLC, San Diego, CA) by referencing the *V. oldhamii* chloroplast genome (MK049537). The circular chloroplast genome map of *V. henryi* was drawn using the OGDRAW tool (Greiner et al. 2019). The cis- and trans-splicing genes were drawn by CPGView (http://www.1kmpg.cn/cpgview/, Liu et al. 2023).

To conduct a comprehensive phylogenetic analysis, complete chloroplast genomes of 17 *Vaccinium* species and two outgroups (*Rhododendron calophytum* and *Gaultheria griffithiana*) were used. All complete chloroplast genome sequences were aligned using MAFFT v7.309 (Katoh and Standley , 2013) and applied to construct a phylogenetic tree using maximum-likelihood (ML) methods with 1000 bootstrap replicates implemented in the RAxML tool (Stamatakis 2014), and the best model (TVM+F + R4) selected from ModelFinder version 1.6 (Kalyaanamoorthy et al^2017^) was applied.

## 3. Results

**Figure 2.**
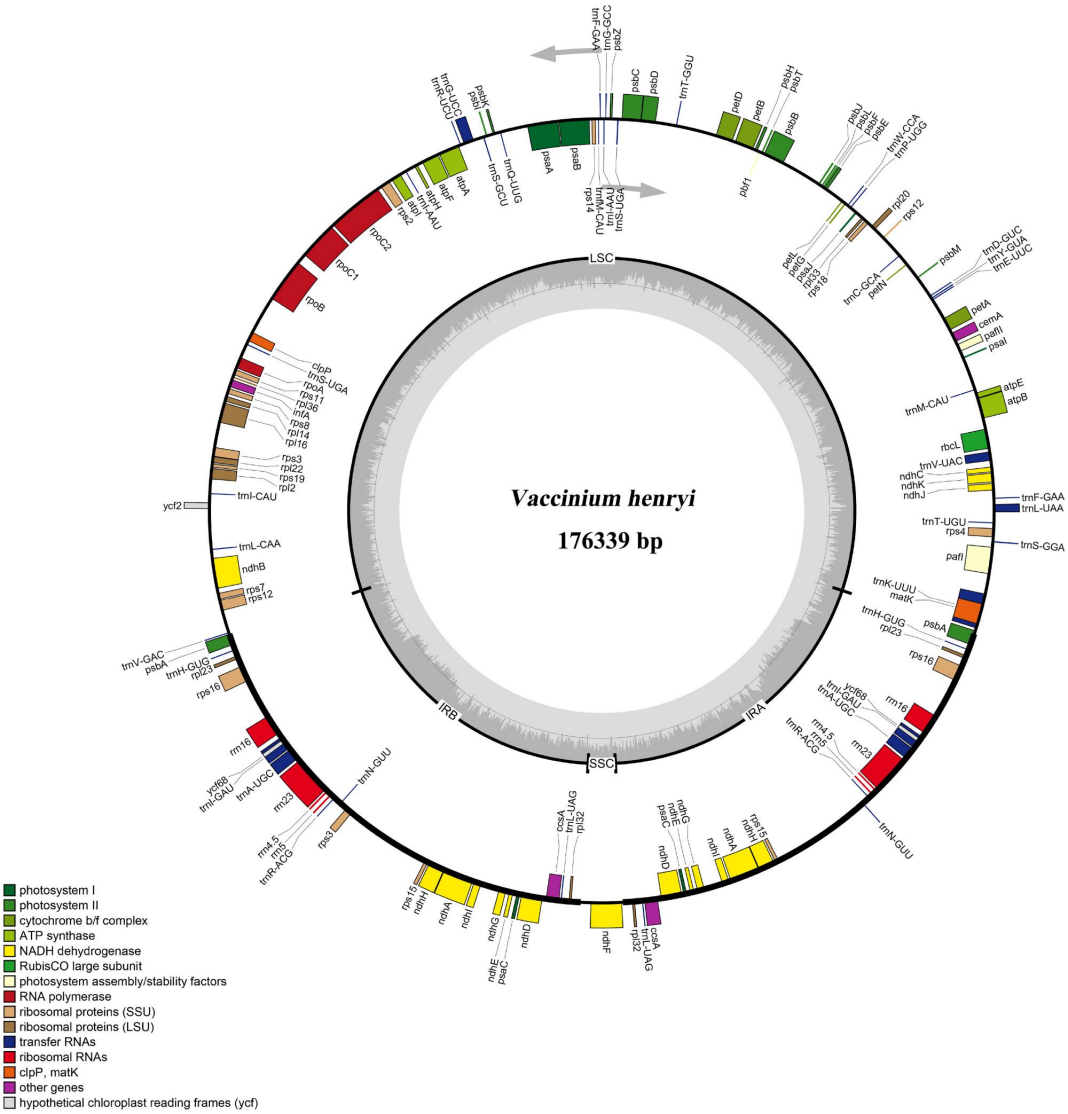
The complete chloroplast genome map of *V. henryi*. Genes lying outside the circle are transcribed in the counterclockwise direction, and those inside are transcribed in the clockwise direction. The quadripartite structure is marked with LSC, SSC, and IRA/IRB regions. Different functional genes are represented by bars with different colors. The relative GC content of chloroplast genome was indicated with inner gray circle.

The complete chloroplast genome of *V. henryi* was 176,339 bp in length, with an overall GC content of 36.77%. The maximum sequencing coverage depth was 4573×, while the minimum coverage was 8×, and the average sequencing coverage depth was 1734.48× (Supplementary Figure S1). The genome consisted of two inverted repeats (IRs) regions of 33,601 bp each, a 106,115 bp large single copy (LSC) region, and a 3022 bp small single copy (SSC) region, the SSC region was relatively small and contained only one gene, namely *ndhF* (Figure 2). Finally, 139 unique genes were annotated, including 91 protein-coding genes (PCGs), 40 tRNA genes, and 8 rRNA genes. Among these genes, 15 genes contained one intron and two genes owned two introns, and 24 genes were duplicated, including 13 PCGs, seven tRNA genes and four rRNA genes (Supplementary Table S1). Besides, the structures of nine cis-splicing genes (*pafI*, *petB*, *petD*, *atpF*, *rpoC1*, *rpl16*, *ndhB*, *rps16*, *ndhA*), and one trans-splicing gene (*rps12*) were also detected (Supplementary Figure S2).

**Figure 3.**
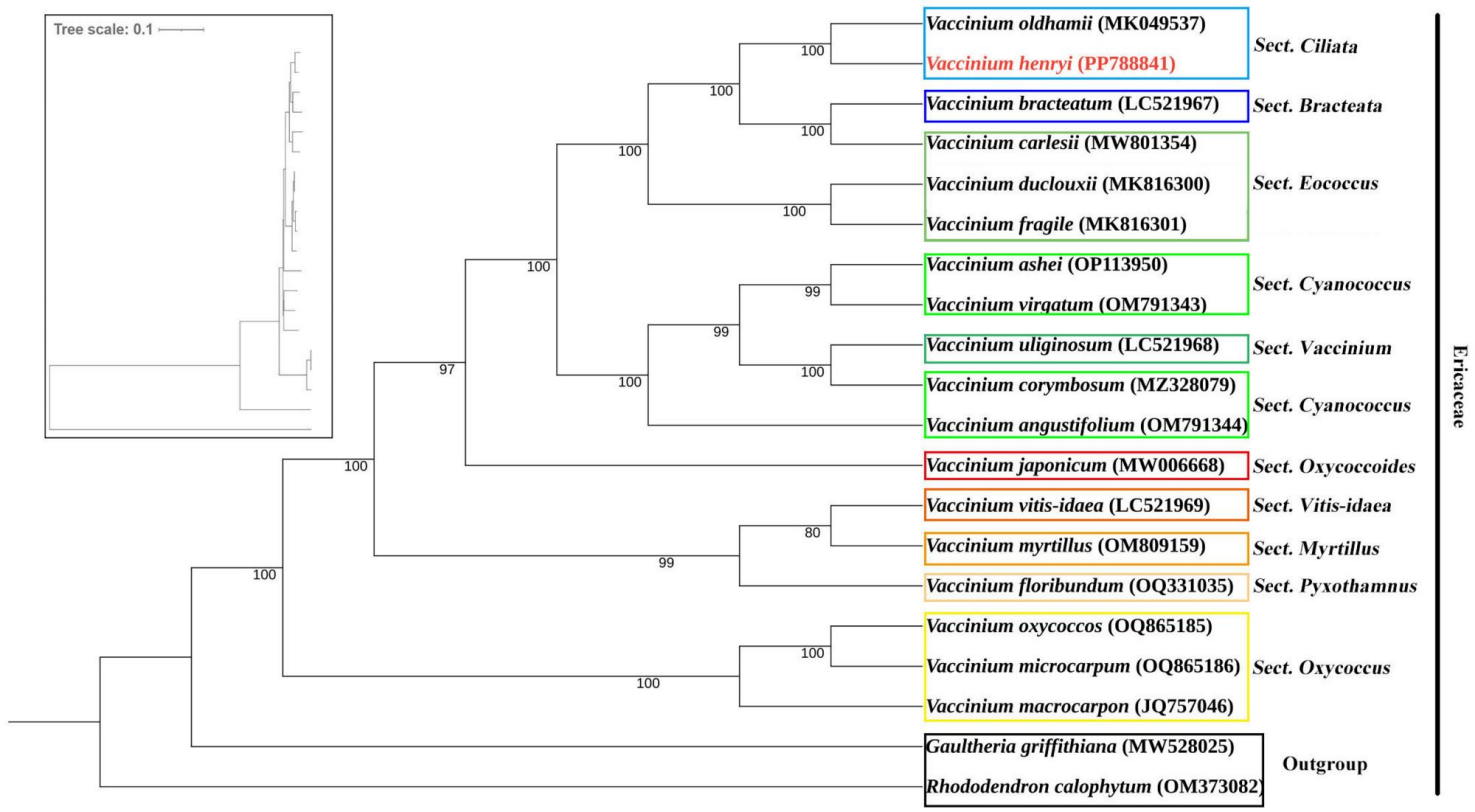
Phylogenetic tree based on complete chloroplast genomes of 20 species from Ericaceae family. *V. henryi* (PP788841) was marked in red, *V. henryi* and *V. oldhamii* belong to section *Ciliata* were marked with blue frame, the remained species marked with different color frames belong to different sections. Branch labels indicated the bootstrap support values. The sequences of following species were used, including *V. corymbosum* MZ328079 (Miao et al. 2022), *V. fragile* MK816301 (Guo et al. 2019), *V. duclouxii* MK816300 (Chen et al. 2019), *V. oldhamii* MK049537 (Kim et al. 2019), *V. bracteatum* LC521967 (Kim et al. 2020), *V. vitis-idaea* LC521969 (Kim et al. 2020), *V. macrocarpon* JQ757046 (Fajardo et al. 2013), *V. japonicum* MW006668 (Cho et al. 2021), *V. uliginosum* LC521968 (Kim et al. 2020), *V. microcarpum* OQ865186 (Fahrenkrog et al. 2022), *V. oxycoccos* OQ865185 (Qiao et al. 2023), *V. ashei* OP113950 (Fahrenkrog et al. 2022), *V. virgatum* OM791343 (Fahrenkrog et al. 2022), *V. angustifolium* OM791344 (Fahrenkrog et al. 2022), *V. myrtillus* OM809159 (Fahrenkrog et al. 2022), *V. floribundum* OQ331035 (Fahrenkrog et al. 2022), *V. carlesii* MW801354 (https://www.ncbi.nlm.nih.gov/nuccore/MW801354.1/), and two outgroup species (*Rhododendron calophytum* OM373082 (Ma et al. 2022) and *Gaultheria griffithiana* MW528025 (Li et al. 2021)). the tree scale indicates the base substitution ratio.

To clarify the evolutionary position of *V. henryi* in the genus *Vaccinium*, an ML tree was constructed with 1,000 bootstrap replicates by using 17 published complete chloroplast genomes of *Vaccinium* species and two outgroup species. The phylogenetic tree (Figure 3) revealed that among the 17 *Vaccinium* species analyzed, several exhibited specific sectional affiliations: *V. henyi* and *V. oldhamii* belong to section *Ciliata*, *V. carlesii*, *V. duclousii* and *V. fragile* belong to section *Eococcus*, *V. ashei*, *V. virgatum*, *V. corymbosum* and *V. angustifolium* belong to section Cyanococcus, *V. oxycoccos*, *V. microcarpum* and *V. macrocarpon* belong to Section *Oxycoccus*, and the rest species belong to individual sections. *V. henryi* was more closely related to *V. oldhamii* among all 17 *Vaccinium* species.

## 4. Discussion and conclusion

In this study, the chloroplast genome of *V. henryi* was *de novo* sequenced and assembled, revealing a comprehensive gene complement of 139 genes (27 duplicates), including 91 protein-coding genes (13 duplicates), 40 tRNA genes (7 duplicates), and 8 rRNA genes (4 duplicates). It shared a similar structure with other *Vaccinium* species. Notably, significant variations were observed in the number of chloroplast genome genes among different *Vaccinium* species, which ranged from 123 to 144 genes (Kim et al. 2019; Fahrenkrog et al. 2022). These differences were predominantly observed in the protein-coding genes and tRNA genes, while rRNA genes remained consistent, 8 genes including 4 duplicates. With limited genomes available, mechanisms underlying the structural variation in the chloroplast genomes of the *Vaccinium* species still need further investigation.

Genus *Vaccinium* L. comprises approximately 450 species that can be classified into over 30 distinct sections (Vander Kloet and Dickinson 2009). Phylogenetic analyses based on chloroplast genome data accurately distinguished and illuminated the relationships of these *Vaccinium* species. In this study, the phylogenetic tree indicated *V. henryi* and *V. oldhamii* were closely related, consistent with the traditional morphological section classification. Given that chloroplast introgression is very prevalent among *Vaccinium* species (Beeler et al. 2020; Zhidkin and Matveeva 2022), and *V. henryi* and *V. oldhamii* are mainly distributed in China, chloroplast introgression may contribute to their close phylogenetic affinity. Additionally, *V. oxycoccos*, *V. microcarpum* and *V. macrocarpon* were clustered within section *Oxycoccus*, *V. ashei*, *V. virgatum*, *V. corymbosum* and *V. angustifolium* were clustered in section *Cyanococcus* as traditional morphological classification. *V. uliginosum* presented an exception, exhibiting close relatedness to section *Cyanococcus* species despite belonging to section *Vaccinium*. It is hypothesized that the high crossbreeding compatibility between *V. uliginosum* and *V. corymbosum* might account for their phylogenetic proximity (Fahrenkrog et al. 2022). Further genetic investigations are still warranted to elucidate this relationship more comprehensively.

In conclusion, we sequenced and assembled the first chloroplast genome of *V. henryi* by using next-generation sequencing. The chloroplast genome would provide us valuable genetic information of *V. henryi*, and knowledge of evolutionary relationships among *Vaccinium* species.

## Supplementary Material

Supplementary Table S1.xlsx

## Data Availability

The chloroplast genome sequence data of *V. henryi* are openly available in the GenBank of NCBI at https://www.ncbi.nlm.nih.gov/ under the accession no. PP788841. The associated BioProject, SRA, and Bio-Sample numbers are PRJNA1109552, SRR29000694, and SAMN41274345, respectively.
